# Anti-Adhesion Behavior from Ring-Strain Amine Cyclic Monolayers Grafted on Silicon (111) Surfaces

**DOI:** 10.1038/s41598-020-65710-w

**Published:** 2020-05-29

**Authors:** Jing Yuan Ching, Brian. J. Huang, Yu-Ting Hsu, Yit Lung Khung

**Affiliations:** 1 Department of Biological Science and Technology, China Medical University, No.91 Hsueh-Shih Road, Taichung, Taiwan; 2 Integrative Stem Cell Center, China Medical University Hospital, Taichung, 40447 Taiwan; 3 Institute of New Drug Development, China Medical University, No.91 Hsueh-Shih Road, Taichung, Taiwan

**Keywords:** Biosurfaces, Surface assembly, Surface spectroscopy, Nanobiotechnology

## Abstract

In this manuscript, a series of amine tagged short cyclic molecules (cyclopropylamine, cyclobutylamine, cyclopentylamine and cyclohexylamine) were thermally grafted onto *p*-type silicon (111) hydride surfaces via nucleophilic addition. The chemistries of these grafting were verified via XPS, AFM and sessile droplet measurements. Confocal microscopy and cell viability assay was performed on these surfaces incubated for 24 hours with triple negative breast cancer cells (MDA-MB 231), gastric adenocarcinoma cells (AGS) endometrial adenocarcinoma (Hec1A). All cell types had shown a significant reduction when incubated on these ring-strain cyclic monolayer surfaces than compared to standard controls. The expression level of focal adhesion proteins (vinculin, paxilin, talin and zyxin) were subsequently quantified for all three cell types via qPCR analysis. Cells incubate on these surface grafting were observed to have reduced levels of adhesion protein expression than compared to positive controls (collagen coating and APTES). A potential application of these anti-adhesive surfaces is the maintenance of the chondrocyte phenotype during *in-vitro* cell expansion. Articular chondrocytes cultured for 6 days on ring strained cyclopropane-modified surfaces was able to proliferate but had maintained a spheroid/aggregated phenotype with higher *COL2A1* and *ACAN* gene expression. Herein, these findings had help promote grafting of cyclic monolayers as an viable alternative for producing antifouling surfaces.

## Introduction

Obtaining good anti-biofouling surfaces via chemical modification is important for many surface chemistry groups due to huge commercial implications in many medical applications, ranging from biosensors to bioimplants. Of the many surface chemistries reported, polyethylene glycol (PEG) based chemical grafting is currently the most popular option to provide anti-biofouling surface on many types of substrates^1–4^ although it is necessary to note that there are also many other interesting chemical alternatives such as zwitterions^5,6^ or amphiphilic polymers^7,8^. Often, the underlying mechanisms of surface grafted hydrophilic PEG conferring anti-biofouling attributes is well-understood and is nominally ascribed as having a strongly bound hydration layer that can resist attachment from incoming proteins^5,9,10^. In terms of the recipient substrate, silicon-based substrate remains an important material for anti-biofouling passivation in view of its diverse applications that may range from biosensing^11,12^ to drug delivery^13^. Furthermore, these materials had often been the candidate of choice for examining interfacial behaviour of cells as the spectrum of surface chemistry can be easily fine-tuned by many surface grafting techniques such as silanization^4,14,15^ or hydrosilylation^16–21^.

However, Si-O-Si linkage produced from silanization may be unstable under aqueous/physiological environment^22,23^. Hence, attaining a more stable surface bonding such as Si-C is often more useful for biological applications. To produce Si-C surface bonding, hydrosilylation is the most common synthetic route as first described by Chidsey *et al*.^24,25^ and the overall reaction is summarized as interaction of a Si-H and an unsaturated carbon chain that would ultimately produce a Si-C surface linkage. This process may be initiated via thermal, catalytic and photon-induced reactions^17,26,27^. While various proposed mechanisms for both catalytic and photon induced reactions are well-established, the mechanism for thermal mechanism remains highly argumentative^28^. Furthermore, one of primary disadvantage for thermal based hydrosilylation is the susceptibility of the reaction towards the presence of electron-rich nucleophiles (OH, NH_2_) and this had greatly limited the choice of compounds that can be used in the grafting process^29^. Interestingly, it is also this susceptibility that had presented newer opportunities in terms of producing novel silicon surface grafting.

In the past few years, our group had focused on describing the susceptibility of nucleophiles to silicon hydride surface^30–34^. In the presence of an amine, thermal based reaction to silicon hydride surface would produce a surface grafting via Si-N linkage^31,34,35^, typically through electron donating NH_2_ forming direct dative bonding to electron accepting silicon hydride as described by Hamer’s group^36,37^. Interestingly, Si-N surface bond may in fact be more stable as the bond dissociation enthalpy for Si-N is 355 kJ/mol while Si-C bond is 318 kJ/mol^38^. When this is compared with Si-Si bonds (222 kJ/mol), it is easy to appreciate how Si-N bonds may represent an increase in stability compared to Si-C and Si-Si bonds on oxide-free silicon surface. Furthermore, Si-N linkage may also confer higher thermal stability that may be extremely useful if the grafting is to be subjected to subsequent thermal reactions.

In one previous study from our group^31^, we reported that thermal reaction of cyclopropylamine to silicon hydrogenated surfaces would passivate the surface via Si-N bond and that no radical was observed to have formed during the thermal reaction. What was more interesting from this study was that surface grafted cyclopropylamine had exhibited a highly unusual characteristic when this monolayer serves as incubating platform for immortalized cancer cells. We had noticed that the ring-strain cyclopropane had consistently impeded proper cellular adhesion and this is both highly intriguing as well as puzzling for the fact that this had not be reported in literature previously. It is also this unusual behaviour of cyclopropylamine grafted monolayer that had provided the necessary impetus for this report.

**Figure 1:**
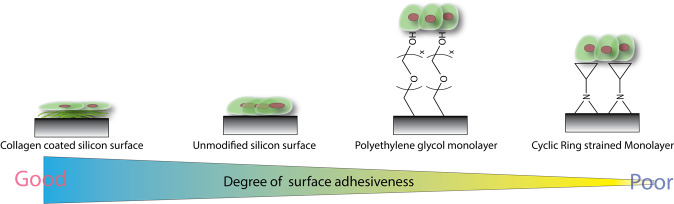
Graphical illustration on the degree of surface adhesiveness relative to the type of surface modification. Interestingly cyclic ring strained monolayers in this work was found to resist cellular spreading on the surface.

As illustrated by Fig. 1, hydrophilic surfaces had been commonly utilized by many groups as means of repelling protein absorption and cellular adhesion. Over the past few decades, the general consensus had been that a strongly bound hydration layer on the surface would be difficult to dislodge to facilitate for the absorption of protein^5,39–41^. Hence, many groups had invested heavily on surface chemistry to create thick hydrophilic polymer graftings on the surfaces in order to produce anti-biofouling effect. In fact, it is widely accepted that surface hydrophilic layer must be sufficiently thick in order to improve the anti-biofouling effect^4,42^. From this standpoint, it is therefore unusual that a thin monolayer of cyclopropane grafting (<2 nm) could provide similar anti-adhesive effect. Certainly, the idea of a superhydrophobic surface may also discourage cell adhesion may be attributed^43,44^ but these previously reported surfaces are usually highly structured and/or under constant fluid drag with a superhydrophobic profile (>150°)^2^. Furthermore, with these surfaces resisting cell/protein adhesion, it is often necessary to have relatively thick organic layer on the surface^45^. But yet, our studies had consistently indicated that we could achieve low anti-biofouling characteristics from silicon substrate thermally grafted with cyclopropylamine. This had strongly suggested that a thin nanolayer of ring-strained cyclic monolayer may have certain anti-biofouling effect that formed the very basis of this current report.

Hence, in order to gain better insights into ring-strained monolayers resisting cell adhesion, we had examined a series of different of different ring-strain cyclic molecules (cyclopropylamine, cyclobutylamine, cyclopentylamine and cyclohexylamine) that were thermally grafted to silicon (100) hydride surface. The surfaces were subsequently examined by high-resolution XPS and AFM to validate the surface chemistry and its physical characteristics. Three different types of cells displaying different morphologies (MBA-MD 231 cells, AGS and Hec1A) were incubated for 24 hours on these surfaces and cell viability/cell count, confocal and fluorescence microscopy were performed to enumerate the adherent cells while qPCR was carried out to quantify focal adhesion proteins ((Vinculin, Paxilin, Talin and Zyxin) with respect to positive controls (collagen and APTES coated silicon). From the studies, we also selected cyclopropylamine grafting to perform a 6 days incubation of primary chondrocytes and quantitative qPCR was also performed to examine the adhesion outcome of these primary stem cells line grown on our modified surfaces.

## Methods and materials

Boron-doped (111) silicon wafers, with resistivity of 0.001–0.005 Ω-cm were acquired from acquired from Semiconductor Wafer, Inc. (SWI, Taiwan) for these experiments. Unless otherwise stated, all reagents were purchased from Sigma-Aldrich and were used as received without further purification.

### Thermal grafting of the cyclic molecules and 1,7 Octadiyne

In a process akin to our previous reports for thermal grafting^31,34,35,46^, silicon (111) wafers of 10 × 10 mm^2^ were first prepared using diamond cutter and subsequently immersed in hot Pirahna solution (3 parts 95% Sulfuric acid and 1 part 34.5–36.5% hydrogen peroxide) for at least 30 mins to remove any residual hydrocarbons and contaminants from the surface. These wafers were then introduced into aqueous solution of 5% hydrofluoric acid for 30 seconds to produce silicon hydrogenated surface. Next, the sample were placed in a customized glass reactor containing degassed (via at least ten freeze-pump-thaw cycles) solution of 100 mM of the various cyclic amine compound (cyclopropylamine, cyclobutylamine, cyclopentylamine and cyclohexylamine respectively) or 1,7 Octadiyne (control) in mesitylene and subsequently stored under argon. The temperature of the reactor was raised to 150 °C in an oil bath and left to reacted for approximately 18 hrs in the dark. After the thermal reaction, the surfaces were rinsed with copious amount of mesitylene to remove any unreacted compound and this was followed by rinse/sonication of methanol, ethanol, dichloromethane and deionised water in quick sequential order. All surfaces were then kept under vacuum prior to use for cell incubation as well as surface characterization.

### Contact-angle measurements

A custom-made goniometer was used to acquire all water droplet profiles on the various surfaces. Comprising of a CCD camera (SMN-B050-U (B/W)) that would acquire images at a resolution of 2560 × 1920, three 2 µl droplets were carefully dispensed on each surface grafting and the image was captured on the software as provided. Post-analysis of the droplet was subsequently performed using the Dropsnake 2.1 add-on feature in ImageJ software bundled with 64-bit Java 1.8.0_112.

### X-ray Photoelectron spectroscopy (XPS)

Unless otherwise specified, all high-resolution x-ray photoelectron spectrograms were acquired on a PHI 5000 VersaProbe (ULVAC-PHI) equipped with an Al Kα X-ray source (1486.6 eV). All spectrums were taken at 45**°** relative to the surface and the analysis was performed at National Chung Hsing University in Taichung. High resolution spectra were obtained for the C1s, O1s, Si2p and N1s in high resolution for all surfaces. The peaks were subsequently analysed and deconvoluted using XPSpeak 4.1 while the atomic concentration was carefully determined with regional binning provided in CasaXPS (version 2.3.18) software

### Atomic force microscopy (AFM)

Atomic force microscopy (AFM) of all surfaces were carried out at Feng Chia University in Taichung. All images were acquired using the Digital Instrument NS4/D3100CL/MultiMode Scanning Probe Microscope software as provided by the developer and surfaces were analysed via AFM tapping mode with the cantilever tuned at Freq. 150 kHz with a Force of 5 N/m. Scan area on the surfaces were all preset at 1 μm x 1 μm and images were digitally scanned at a scan rate of 0.8 hz with the automatic integral and proportional gain setting. Post image processing were performed using Gwyddion MacOS version 2.38.

### Surface silanization for APTES and PEG

Unmodified silicon (111) wafers were first treated with Piranha solution and subsequently washed with deionised water. The silicon surfaces were then introduced to 50 mM of APTES in toluene at room temperature for a duration of 2 hours. After the reaction, the silicon wafer was washed in methanol, ethanol and dichloromethane (DCM) followed by deionised water rinsing for 1 minute. Prior to use in cell culture studies, APTES functionalized surfaces were also sterilized by first rinsing the surfaces with a filter-sterilized 70% ethanol followed by UV light exposure of 30 minutes. For pegylation, 50 mM of 2-[methoxy(polyethyleneoxy)6-9 propyl]trimethoxysilane (Gelest) in ethanol was used under similar experiment conditions to those for APTES as described earlier.

### Collagen grafted surface

Silicon surfaces were first clean in Piranha solution for 30 mins and were then rinsed wash with deionised water before being blown dry with argon. The surfaces were then sterilized by exposure to UV light for 30 mins. After that, 200 μl of 0.1% collagen (Type 1 from rat tail, Sigma-Aldrich) was gently added to the silicon surfaces and the surfaces were left to dry for 30 minutes in a laminar air-flow. Subsequently, excess fluid on the surface was carefully drained away and the coated surface were subsequently left to be dried at room temperature before use.

### Cell culture

In a fashion similar to our previous report^35^, triple negative breast cancer MDA-MB-231 cells (Cell BRCR number: 60425) and Human gastric adenocarcinoma AGS cells (Cell BRCR number: 60102) were cultured in RPMI media that were supplemented with 10% fetal bovine serum and 1% penicillin−streptomycin. Uterus endometrium adenocarcinoma HEC1A cells (Cell BRCR number: 60552) were cultured in DMEM F12 supplemented with 10% fetal bovine serum and 1% penicillin−streptomycin. The various silicon samples (10 mm by 10 mm) were carefully positioned in a 24-well plate and seeded with initial density of 3 × 10^4^ cells for 30 minutes. Subsequently, non-adhering cells floating in the medium was removed by gently washing the surfaces with phosphate-buffered saline (PBS) in order to avoid cellular aggregation that could potentially affect subsequent analysis. Incubating cell medium was quickly replenished into these wells and the cells were incubated in a 5% CO2 incubator at 37 °C for a duration of 24 hours. As a proof of concept, commercially available Human Mesenchymal Stem Cells (MSC) acquired from Celprogen Inc (Catalog Number: 36094-21) were cultured for a period of 7 days on unmodified, cyclopropylamine grafted silicon surfaces as well as on collagen coated surfaces. 2 × 10^4^ were initially seeded in 500 μl of Celprogen undifferentiated media on 1 × 1 cm silicon substrates and the media was replenished every 3 days of the culture. All cell culture on the surfaces had been performed in triplicates unless otherwise stated.

### Cell viability, cell count and cytotoxicity studies

In a protocol similar to our previous report^35^, after 24 hours incubation, all surfaces were carefully introduced into new 24-well plate and 200 μL of cell medium and 40 μL of assay solution (Cell Meter Colorimetric Cell Cytotoxicity Assay Kit purchased from AAT Bioquest) was added to each of these wells. The solutions were carefully mixed by gently shaking for 30 seconds. The surfaces were incubated again at 37 °C in the CO2 incubator for 4 h. After the incubation time, the absorbance change in the 24-well plate was determined at 570 and 605 nm from a multiplate reader. The ratio of OD570 to OD605 was used to quantify the cell viability from each well. The readings of five replicates were collected, and the values were subsequently normalized at 100% relative to unmodified control (piranha-cleaned) silicon surfaces. Statistical analysis of cell count differences on the various surfaces was derived via a one-way ANOVA analysis with relation to the positive control collagen whereby P value of ≤ 0.05 was taken as statistically significant.

For cell count analysis as shown in Supplementary Figs. S7 and S8, a total of n = 3 surfaces (10 mm by 10 mm each) were selected for each of the surface reaction conditions. Five randomized spots were selected on each of the surfaces and fluorescence microscopy images were acquired on each of these spots at a 10x magnification. The total number of cells on each of these spots was subsequently quantified by manual counting and their values were tabulated with standard derivations with an Excel spreadsheet.

Cytotoxicity studies on 10 mm × 10 mm silicon surfaces grafted with cyclic monolayers had been performed by first attaching these surfaces on tight customized PS cell culture flask with manually premade indentation that would only hold and exposed the silicon surface but not the underlying polystyrene substrate. MDA-MB 231 cells at a quantity of 3 × 10^4^ was then aliquoted carefully on each of the respective surfaces and these cells can only be solely incubated on the silicon surfaces while not ‘spilling’ over to surrounding PS substrate. Subsequently, these surfaces were incubated for 24 hours before MTT was performed to check on the cell viability and to evaluate the toxicity.

### Preparation for confocal microscopy

Similar to our previous report^35^, to examine the cell morphology under confocal microscopy after 24 h of incubation, cells on each surface were fixed in 4% paraformaldehyde for 20 min. A 1 μL stock solution of phalloidin-488 (AAT Bioquest) was diluted in 100 μL of PBS containing 1% bovine serum as the main working solution for staining. The surfaces were also washed with PBS for three times before staining the nuclei with 1 μL of Hoechst 33342 (AAT Bioquest) in 1 mL of PBS buffer for 10 min. Upon completion of staining and washing, a drop of Fluoromount (NOVUS) was carefully aliquoted onto the surface and subsequently covered with a coverslip and sealed with nail varnish. The stained cells with actin filament were observed under confocal microscopy (LEICA SP8) at 40x magnification to examine the cell morphology.

### RNA extraction and RT-qPCR

Cells on each surface were first trypsinized and pelleted followed by washing with PBS. RNA was extracted using RareRNA reagent (Gene Pure, GPR02) in accordance to the manufacturer’s instructions. RNA concentration was then determined by Micro-volume Spectrophotometer (Merinton SMA6000). RT q-PCR was performed using a One-Step RT-PCR Kit (Cat. No.: RP01-II-250) under the Applied Biosystems QuantStudio 3 Real-Time PCR System, 96-well machine. The gene expression levels were quantified using the following primers: Vinculin primers (for: 5′-AAACACAGTTACACTTGTGCACCC-3′, rev: 5′-AACAGAGGGAAGTGTCCCCT-3′), Zyxin primers (for: 5′-AGGCAGAATGTGGCTGTCAAC-3′, rev: 5′-GGTGAAGCAGGCGATGTGG-3′), Talin 1 primers (for: 5′-CCCTGATGTGCGGCTTCG-3′, rev: 5′-TGTCCTGTCAACTGCTGCTTC-3′), Paxillin primers (for: 5′-CCCTGACGAAAGAGAAGCCTAAG-3′, rev: 5′-AGATGCGTGTCTGCTGTTGG-3′). Uisng Beta actin as internal control (for: 5′-ACACTGTGCCCATCTACGAGGGG-3′, rev: 5′-ATGATGGAGTTGAAGGTAGTTTCGTGGAT-3′). The gene expression levels were subsequently calculated using the delta-delta Ct method. Ct values were determined as mean of triplicates.

### Chondrocyte isolation, culture on Si substrates, and gene expression analysis

Primary articular chondrocytes (ACs) were isolated from the femoral condyles and trochlear groove of 2-year-old Lanyu minipigs (Pig Model, Miaoli, Taiwan) of mixed genders. No live animals were used in the present study, as all animals were sacrificed for purposes outside the present study. Cartilage was minced to 1 mm pieces, digested in 0.2% collagenase type 2 (Thermofisher) + 3% FBS (Gibco) in chondrogenic medium (CM), which is comprised of DMEM (high glucose, no glutamine, no pyruvate, Hyclone), 100 nM dexamethasone (Sigma), 1% penicillin/streptomycin/fungizone (Sigma), 1% insulin-transferrin-selenium (Corning), 1% nonessential amino acids (Gibco), 1% GlutaMax (Gibco), 100 mg/mL sodium pyruvate (Sigma), 50 mg/mL ascorbate-2-phosphate (Sigma), and 40 mg/mL L-proline (Sigma) for 18 h on an orbital shaker (55 rpm) in a cell incubator. The cell suspension was then filtered through a 70 µm nylon mesh, washed 3x in CM, and frozen until use. ACs were passaged one time (P1) by seeding cells to T75 flasks at 10,000 cells/cm^2^ in CM + 10% FBS + 10 ng/mL FGF-2 (Peprotech) and culturing for 10 days (medium changed every 3 days). P1 ACs were then seeded to 1 × 1 cm modified Si substrates (i.e., unmodified Si, collagen-functionalized Si, and cyclopropylamine-functionalized Si) at 10,000 cells/cm^2^ in 12-well plates and cultured for 6 days (medium changed once at day 3). Afterwards, cells on substrates were stained with phalloidin (without DAPI, n = 3), as described above, or processed for RNA extraction (n = 3). RNA was extracted with RNAzol (Molecular Research Center) following manufacturer’s protocol and assessed with qRT-PCR.

## Results

### X-ray photoelectron spectroscopy (XPS) analysis and atomic force microscopy (AFM) profiling

**Figure 2:**
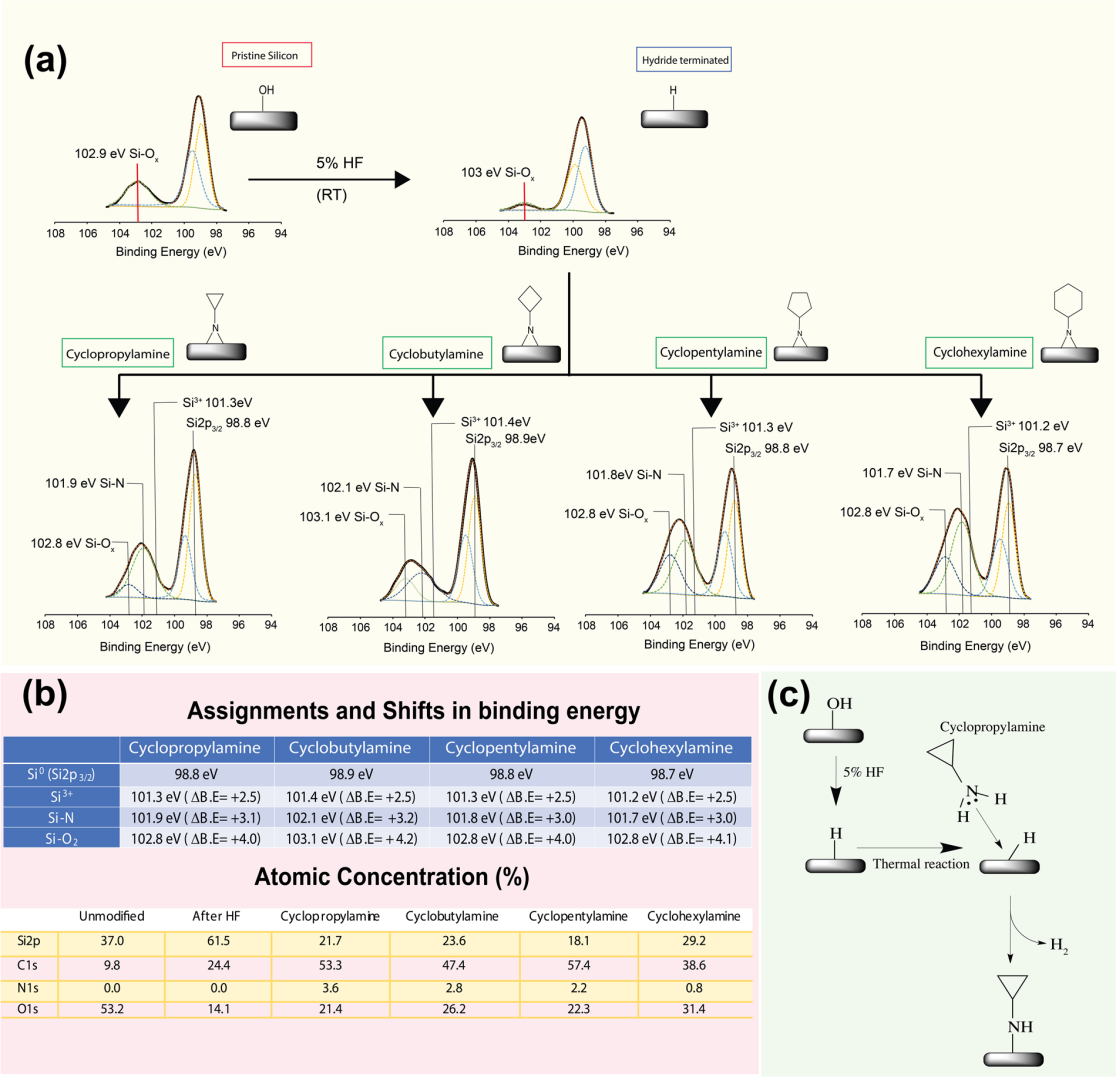
(**a**) Representative spectrums of high-resolution XPS Si2p, N1s and C1s analysis for strained bond modified Si (111) surfaces. The Si2p spectrums had exhibited maximum peak at 101.9–102.1 eV (Si-N) for the modified surfaces contrary to the 103 eV for Si-O on unmodified surfaces, thus suggesting the consistent formation of an Si-N linkage under thermal reaction. (**b**) Shift in binding energy as well as the atomic composition on the surfaces. (**c**) schematic representation of the nucleophilic reaction of cyclopropylamine to silicon hydride surface as discussed in past literature^31,37,48^.

As mentioned in previous sections, four different amino-tagged cyclic molecules were thermally grafted to silicon (111) hydride surfaces. Under the similar conditions, we had previously reported that cyclopropylamine would reacted to the silicon hydride surface via the nucleophilic addition of the amine to the silicon hydride and the observation made in this work was no different (Fig. 2b). It is also important to reiterate that unlike well-documented photoexcitation chemistry on silicon hydride surfaces by which surface radicals help direct the reaction^47^, grafting of cyclopropylamine towards Si-H surface was thought to proceed in nucleophilic fashion via direct electron lone pair interaction from the NH_2_ groups (Lewis base) to form Si-N linkage with silicon serving as lone pair acceptor (Lewis acid), as shown in many previous studies from both other groups^36,37,48–51^ as well as from our previous findings^21,30–34,46^. Furthermore, the solvent mesitylene has relatively low dielectric constant and thus not directly involved in the surface reaction unlike the reaction model as proposed by Ciampi *et al*.^18^. The purpose of p-type substratum selected rather than using n-type silicon was also due to the fact that the current proposed chemistry had already been reported previously for the same series of cyclic amines and thus the nature by which the monolayers are formed had been validated.

The demarcation for oxidation on the grafted silicon was first established from Si2p analysis of unmodified silicon (111) surfaces treated only with piranha solution prior to analysis and we had fixed the bulk Si2p_3/2_ binding energy at 98.6 to 98.9 eV based on our analysis of the high-resolution Si2p spectrum. From Fig. 2a, pristine oxidised surfaces exhibited a peak at 102.9 eV and the distance between the bulk Si2p_3/2_ and the Si-O peak (ΔB.E) was + 3.9 eV and this was in agreement with Si^4+^ oxidation states widely reported in literature^52–54^. On the basis of this, all subsequently Si-O_x_ peaks had therefore been automatically assigned with the same approximation from the bulk Si2p_3/2_ binding energy state. Upon HF treatment for the removal of the thin oxide layer on the surface and the exposure to air for 24 h, slight re-oxidization had occurred at room temperature had produced a Si-O_x_ peak that was found to be at ~4 eV relative to bulk Si2p_3/2_. This had suggested that the recombination of the oxide on the silicon (111) hydride surface was predominantly through Si^4+^ as described by Webb *et al*.^55^. Furthermore, considering that the line constant rate for Si (111) is relatively high due to the atom surface density, it is highly probable the oxidation would proceed a lot faster on these surfaces^56^. Using cyclopropylamine as a reference point for all our subsequent analysis, upon thermal grafting to the Si (111) hydride surfaces, Si2p analysis (Fig. 2a) had displayed a peak centered at 102 eV. Without deconvolution, the shift in binding energy (ΔB.E) from bulk Si2p_3/2_ was estimated to be between 2.9–3.1 eV and incidentally, this peak had fell between the Si^4+^ oxidation states (ΔB.E = + 3.9) and Si^3+^ oxidation states(ΔB.E = + 2.5). On the basis of these observations, we were able to rule out oxidation as the main contributory factor at 102 eV. In fact, during the deconvolution process, we were unable to forcefully curve fit the Si^3+^ oxidation states into our spectrum. Since it was possible to deconvolute peak for Si^4+^ oxidation in the same spectrum, we were more inclined to accept that the thermally grafting of cyclopropylamine would present a mixture of both Si-N and Si-O species, which would subsequently be an important aspect in explaining some of the anti-biofouling characteristics for this work in the discussion section. This was also similar to previous observations made for silicon oxynitride or silicon nitride surfaces that had presented ΔB.E at approximately 2.7–3.0 eV^57,58^. Interestingly, we had also noticed that the largest of the four molecules, cyclohexylamine, was slightly less efficient in grafting to the surface as compared to the other molecules from N1s levels derived off the survey spectrum. This may be in part due to the fact the larger distal 6-carbon ring that may impose greater steric hindrance and therefore reducing surface packing density. In conjunction to Si2p analysis, we were able to elucidate for the peak at representatives 399.1 eV and 401.1 eV representing Si-N for the N1s for all the surfaces (see Fig. S1). These had also helped to confirm that the nitrogen from the distal amine was involved in the nucleophilic reaction to the silicon hydride surfaces as this position is consistent with previous reports on Si-N linkages^59–63^. As there was an absence of unsaturated carbon system in the grafting, only nucleophilic addition of the NH_2_ to the silicon (111) hydride surface was possible as discussed earlier. We had also examined the C1s to confirm for the presence of the C-N bond at 286 eV (see Fig. S2)

**Figure 3:**
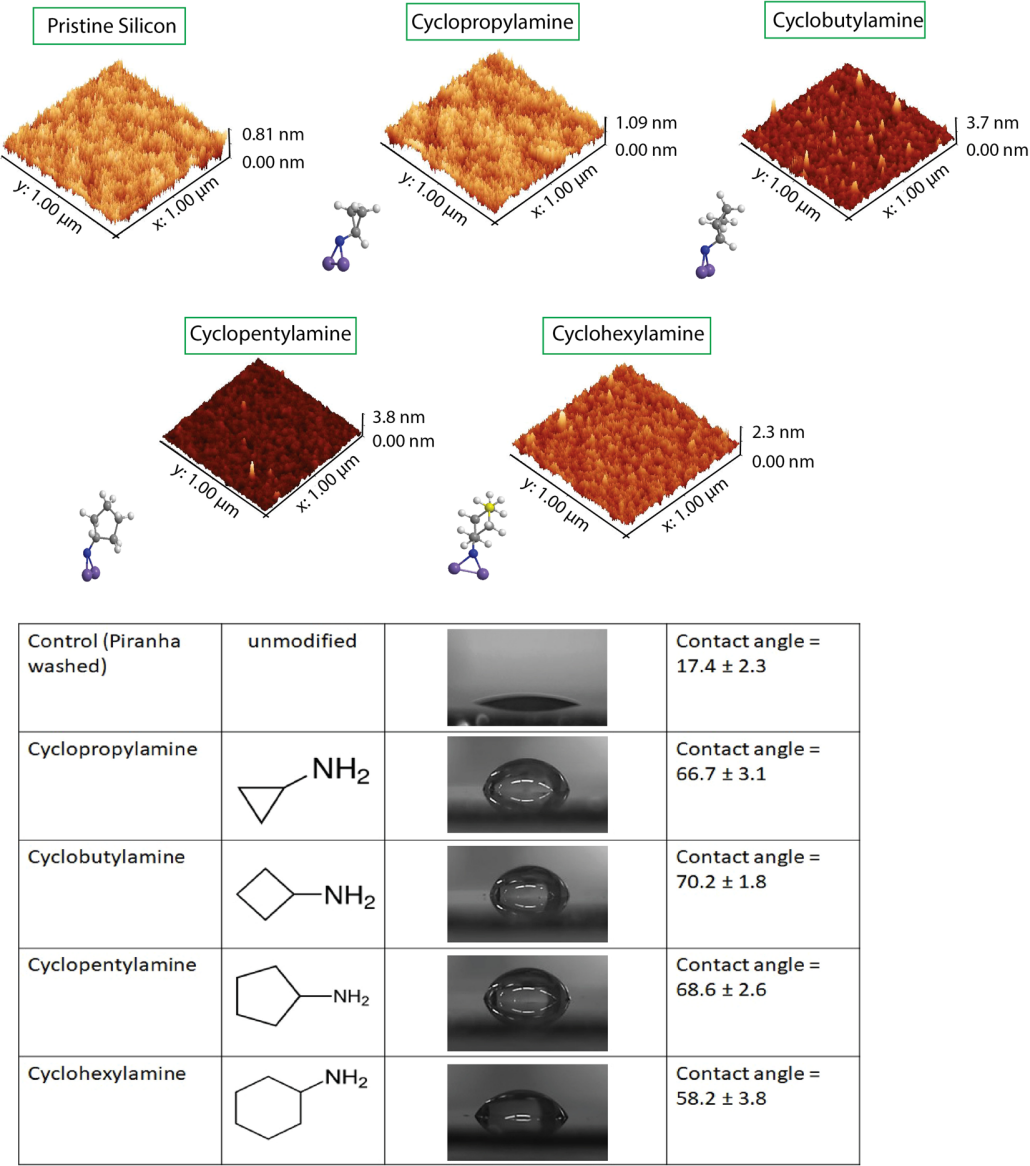
Atomic Force Microscopy of all the cyclic monolayer modified Si (111) surfaces. Their respective contact angle measurements were as tabulated below.

Recovered N content from the survey spectrum (as shown in Fig. 2b) had also helped to confirmed the grafting on the surfaces and it is important to note that while the N content was comparable across all the modified surfaces, the N level for cyclohexylamine was actually lower compared than the other three cyclic molecules. This was mainly attributed to the six-ring member cyclohexane that induce steric hindrances that may subsequently reduce the overall grafting density of the molecule on the surface. From the contact angle measurements, it is possible to see that the thermal grafting on the silicon (111) surface did not result in any significant roughness changes compared to the unmodified silicon surface (Figs. 3 and S5d). This was highly encouraging as the chances of a roughened surface influencing the outcome of subsequent cellular adhesion studies was minimal. The lowering of the packing density of the cyclohexylamine, as previously mentioned, was also found to have an effect on its’ wettability profile (58.2° ± 3.8°) as shown in Fig. 3. However, in view of the similarity in terms of wettability profile for all the grafted molecules as well as the AFM height profiling for cyclopropylamine, cyclobutylamine and cyclopentylamine (ranges between 67°–70°), it was concluded that the nature of the surface grafting was relatively similar and that we had produced a single monolayer on the surface. Interestingly, these wettability profiles may also be some of the main reasons for the subsequent anti-adhesion effects as described by Arima *et al*.^64^ that would be covered in detail in the discussion section of this report. On the other hand, PEG, APTES and collagen surfaces were found to be relatively hydrophilic as shown in Fig. S5a–c.

### Cell-surface viability and confocal microscopy studies

Three different cell types were incubated on our modified Si (111) surfaces. The selection for these cell types (MDA-MB 231, AGS and Hec1A) was due to their distinctive cell morphological shape under normal culture conditions. For instance, healthy MDA-MB 231 cells typically adopt a “needle-like” cell morphology^65^ while AGS cells normally present a polygonal shape while adhering to surfaces^66^. On the other hand, Hec1A is often roundish in morphology without any distinctive polarity or directionality and are not very highly adherent cells^35,67^. The emphasis on cellular morphology was necessary in our attempt to understand how the cells interface with the surface and how the cell adhesion may be affected on our modified surfaces.

**Figure 4:**
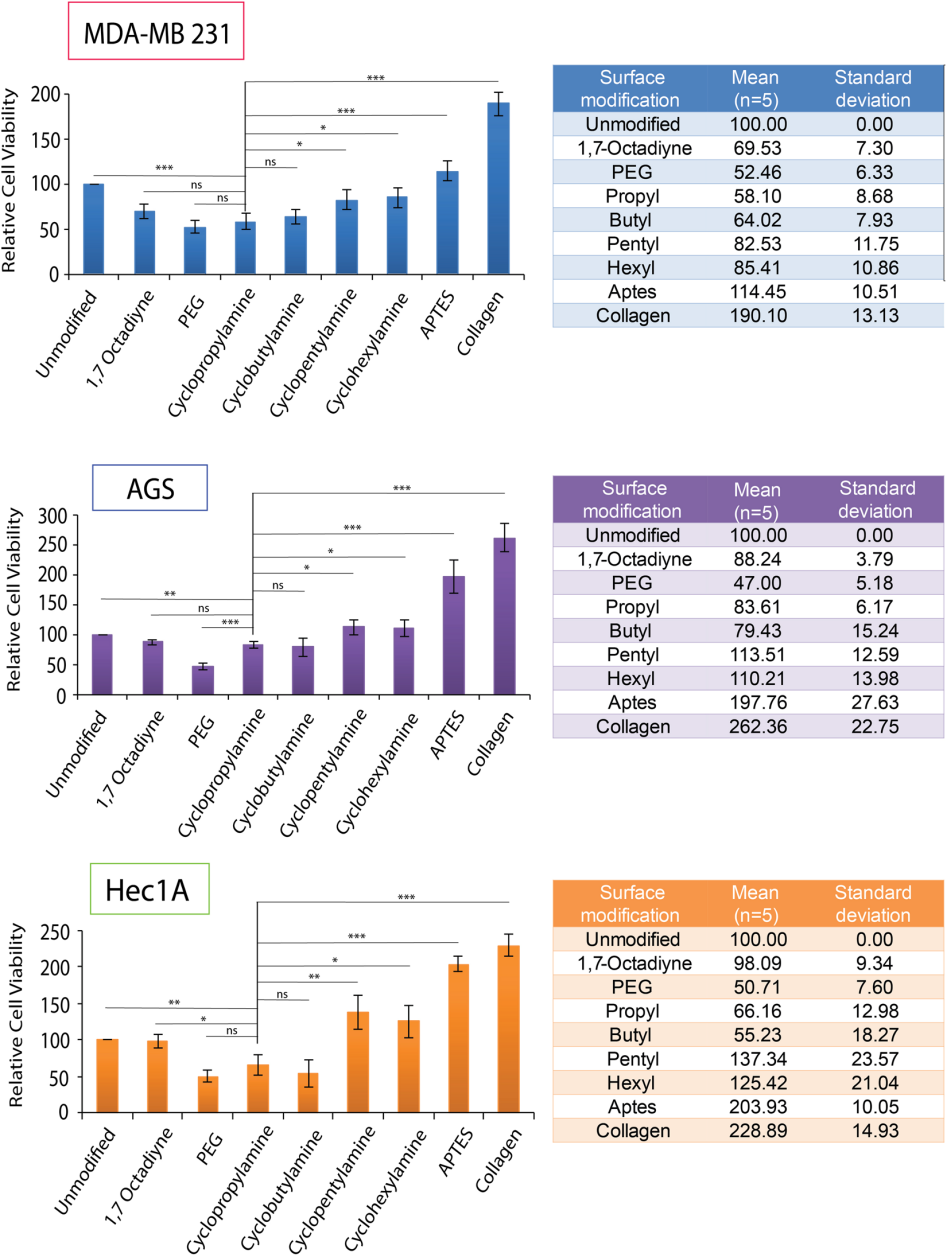
Cell viability assay for MDA-MB 231, AGS and Hec1A cells on the various surface modified substrate after 24 hours of incubation time. Statistical significance shown here is denoted as follows: * represents a *p* value ≤ 0.05, ** represents a *p* value ≤ 0.01, and *** represents *p* value ≤ 0.001, while NS represents a value not statistically significant.

Right from the onset with cell viability assay, we quickly realised that silicon (111) surfaces grafted with the cyclic monolayers were highly anti-biofouling in nature and only a few cells had been observed microscopically after washing. This had resulted in a lower cellular number as well as low cellular viability as shown in Fig. 4. On the other hand, positive surface controls promoting cell adhesion such as APTES, and collagen had shown significantly higher levels of measured cell viability and this was within expectation. In conjunction to the cyclic monolayers, 1,7 octadiyne was also grafted to the silicon surface to serve as a comparative representative as aliphatic self-assembled monolayer. Yet interestingly, for the cyclic based monolayers, we had observed lower cellular viability for all three cell types that was highly comparable to our PEG grafted negative control. Cell count data collected correlated with the observed cellular viability values and a high level of consistency for the cell viability (with low standard derivation) for all ring-strained cyclic monolayers was quickly established. In fact, the levels of cellular viability on ring-strained cyclic monolayers were also consistently lower than on surfaces grafted with 1,7 octadiyne serving as one of the controls. The results had suggested that such monolayers may serve effectively as anti-biofouling grafting and the results were highly comparable to the control PEG grafting, especially for cyclopropylamine and cyclobutylamine. When the values on cyclopropylamine grafted surface was compared against unmodified silicon, we found the anti-biofouling effects was statistically significant for all cell types, especially for MDA-MB 231. Furthermore, we had also decided to perform toxicity test of these chemistries by culturing MDA-MB 231 cells on cyclic-monolayer grafting without washing for 24 hours and there was no notable reduction in cellular viability compared to collagen and APTES (Fig. S3). This had suggested that there was no noticeable cytoxicity from these surface modifications.

**Figure 5:**
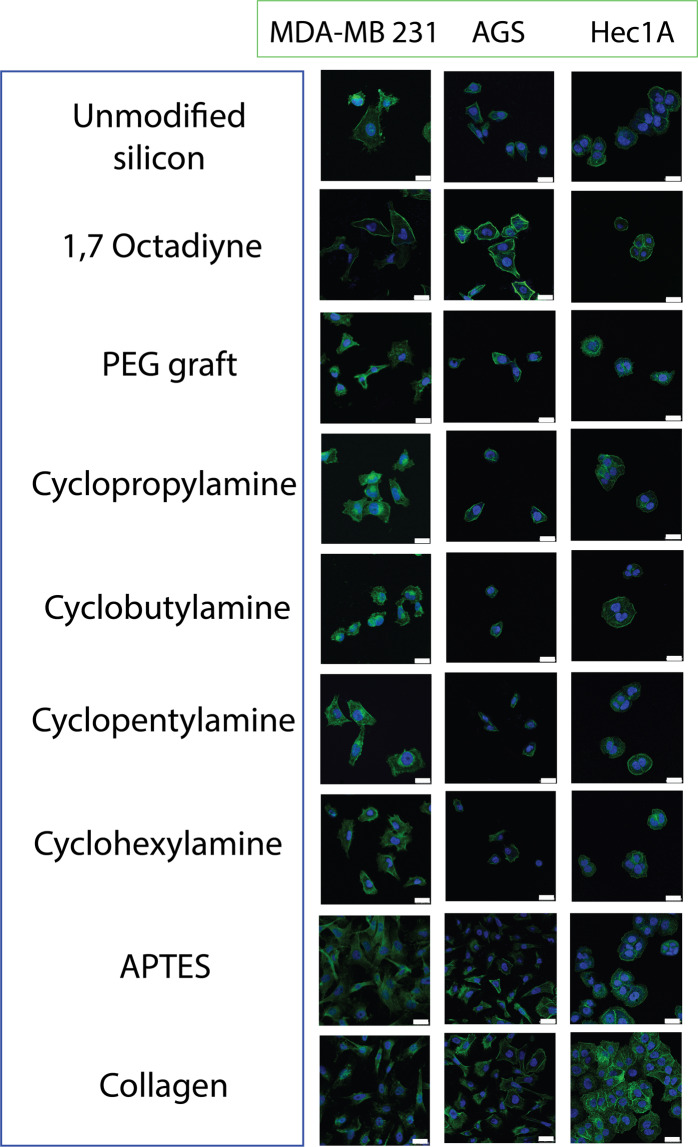
Confocal Microscopy images of the MDA-MB 231, AGS and Hec1A cells on the various modified surfaces after 24 hour of cell culture. White scale bars represent 20 μm.

As mentioned earlier, three different cell types were selected on the basis of their different presenting morphologies and thus their adhering morphologies on the surface modified silicon would serve as key observational markers. However, it is important to reiterate again that most of the cells on cyclic monolayers would generally dislodged during our washing procedure and our confocal microscopy was typically performed on the very few cells still found adhering on the surfaces. As shown in Fig. 5, MDA-MB 231 cells that were normally thin and ‘needle-like’ was adversely affected by cyclic monolayers and were found to be more ovalish in their appearance after 24 hours of incubation, especially for cyclopropylamine and cyclobutylamine. More importantly, on 1,7 octadiyne surfaces, they were observed to be able to retain their distinctive cell directionality. On the other hand, while AGS had seemingly lost its polygonal shape and there was no observable difference in cellular morphology for the Hec1A. A graph comparsion (Fig. S6) was made on the relative cell spreading area (compared to unmodified surfaces) and it was clearly obvious that MDA-MB 231 was found to be most affected by the cyclic monolayers. AGS spreading area was also affected by the cyclic monolayers although it was observed that APTES grafting didn’t help promote cell spreading on the surface. Nonetheless, collagen coated surfaces were highly effective in terms of promoting AGS cell spreading and this was within our expectations. Finally, there was very little correlation between cell spreading for Hec1A for all the different surfaces and in view that these cells were generally rounder in morphology, this can be considered as normal although the cell viability and numbers were substantially lower on the ring-strained cyclic monolayer surfaces. A 24-hour FBS incubation and quantification of the protein levels via XPS N1s level had also shown similar trending (Fig. S4)

### Focal adhesion protein expression levels

**Figure 6:**
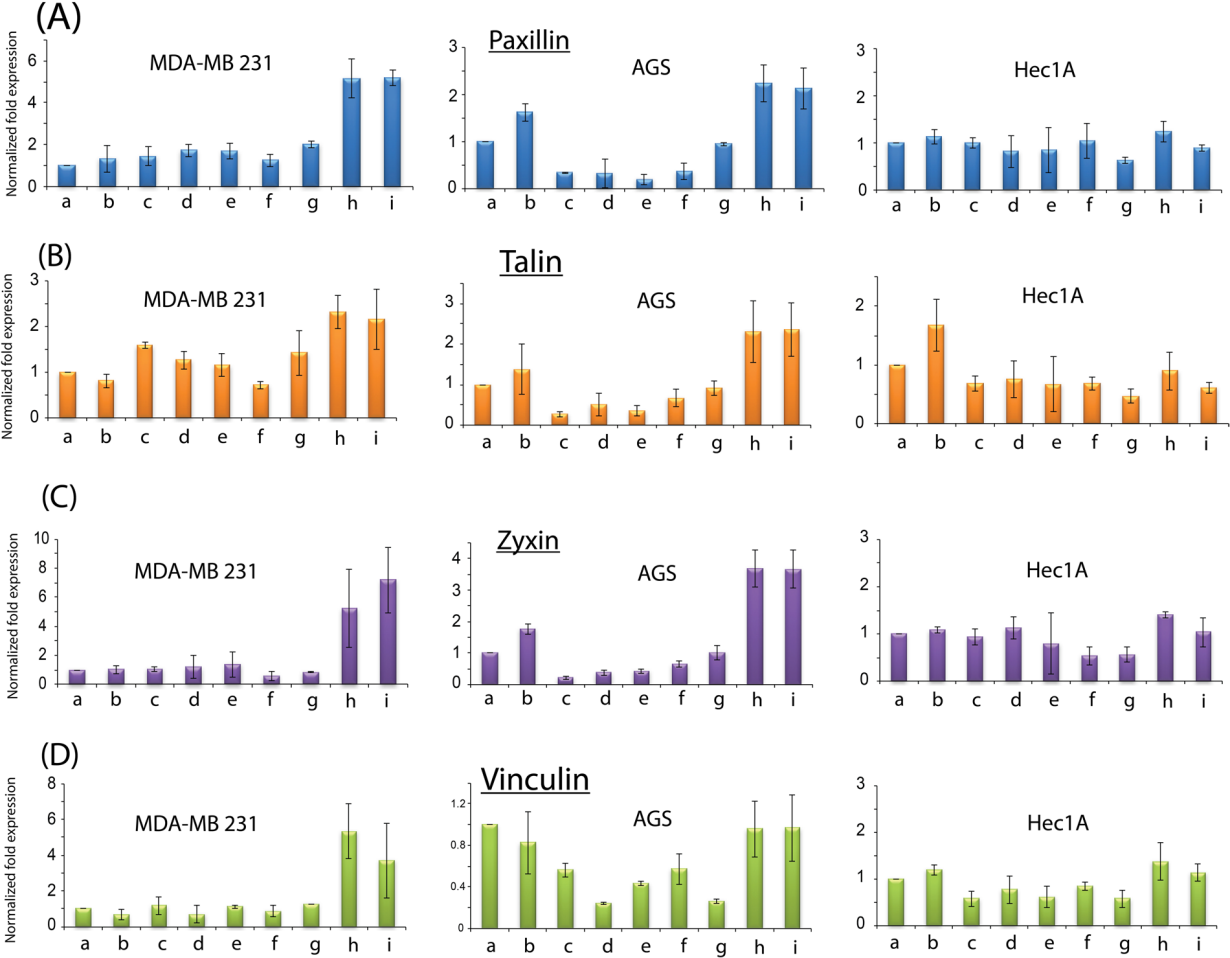
Quantification of the focal adhesion protein expression via q-PCR for (**A**) Paxillin, (**B**) Talin, (**C**) Zyxin and (**D**) Vinculin for MDA-MB 231, AGS and Hec1A cells respectively. All data had been normalized with respect to the unmodified control surfaces. The representations on the x-axis are as follows: (a) unmodified, (b) 1,7 octadiyne, (c) PEG, (d) cyclopropylamine, (e) cyclobutylamine, (f) cyclopentylamine, (g) cyclohexylamine, (h) APTES and (i) collagen coating.

Based on the above results, one hypothesis was that grafting cyclic monolayer on silicon surfaces may discourage the formation of proper focal adhesion points on the surfaces. In order to prove the argument, four classical focal adhesion proteins (Paxillin, Talin, Zyxin and Vinculin)^68–70^ were chosen for quantification via q-PCR after 24 hours of incubation so as to measure transmembrane focal adhesion levels. As shown in Fig. 6, the level of integrin mediated signalling Paxillin was appreciably lower for the cyclic monolayers for MDA-MB 231 and AGS cells as these cells had exhibited distinctive changes to their morphologies (see previous section) while Hec1A was consistent for all surfaces. In fact, from our observations, all four proteins did not show any significant changes in levels for Hec1A regardless of the surfaces type.

Talin’s role for organizing cellular directionality is crucial for directionality on adhering cells and the q-PCR results had suggested that there was a slight depression for MDA-MB 231 cells for the cyclic monolayer compared to the collagen and APTES control. Interestingly, the degree of reduction in gene expression was highly comparable to the values recorded for our negative PEG control. On the other hand, the reduction was more profound for AGS cells when Talin level was compared with the positive control. For Vinculin (Fig. 6D), the levels of gene expression were significantly and statistically lower than positive controls for MDA-MB 231 cells while AGS cells had presented certain fluctuations across the surface modification including the negative PEG. Considering that Vinculin is necessary to maintain cellular shape^71^, it was highly probable that AGS cells were polygonal in shape to begin with and was not as elongated compared to MDA-MB 231 and therefore vinculin expression levels were not as high right on from the onset as illustrated by the magnitude from x-axis between MDA-MB 231 and AGS (Fig. 6D). Zyxin regulates cellular motility^72^ and as both MDA-MB 231 and AGS are highly invasive cancer cell types, there was a huge reduction in gene expression levels for both the negative control as well as the cyclic monolayers while collagen and APTES controls were significantly higher (Fig. 6C).

Based on the q-PCR analysis, we had confirmed that the grafting of the ring-strain cyclic monolayers may impede proper formation of focal adhesion on the surface and the effects were akin to classical anti-biofouling PEG. Collectively, cell viability, confocal microscopy, and qPCR presented a picture that strongly suggested that cyclic monolayers can act as a viable anti-adhesion thin film on silicon surfaces. Furthermore, this effect was not extended to surfaces passivated with long chain aliphatic carbon (1,7 octadiyne) thermally grafted to silicon (111) hydride but was found to be only restricted to ring-strain monolayer systems. This unique anti-adhesion effects of ring-strain cyclic monolayers may be in part due to the higher steric constraints that discourages proper focal adhesion formation on the surface.

### Primary articular chondrocytes culture

Other than potential antifouling applications of these surfaces, changes to cell adhesion through the reduction of talin, paxillin, zyxin, and vinculin mediated signalling may have beneficial applications in controlling cell differentiation. In regenerative cell therapy, cell expansion on tissue culture plastic is an almost universal step to achieve sufficient cell numbers for clinical efficacy, but such an artificial environment is known to induce supraphysiological levels of focal adhesions in expanded cells, which may adversely affect its phenotype. For example, serially passaged chondrocyte on 2D surfaces undergo dedifferentiation, which is marked by a transformation to fibroblast-like morphologies, increase in actin stress fibers, increase in focal adhesions, and decrease in cartilage matrix production (i.e., type II collagen and aggrecan)^73,74^. Direct inhibition of FAK in passaged chondrocytes was shown to induce the loss of fibroblast-like traits and recover levels of type II collagen, aggrecan, and SOX9^75,76^. In view of these issues, several groups had attempted culturing chondrocytes on antifouling surfaces so as to restrict the degree of dedifferentiation. As early as 2004, Mahmood *et al*. had reported on the conservation of chondrocytes on PEG containing polymeric substrates^77^. In recent years, Harata *et al*. had reported on growing chondrocytes on hydrophilic antifouling poly(2-hydroxyethyl methacrylate) surfaces so as to improve on the yield of chondrocytes during harvesting^78^ while Otsuka *et al*. had maintained the nature of chondrocyte spheroids using PEG hydrogel^79^. Cao *et al*. had also examined the effects of micropatterning antifouling PEG surfaces with RGD so as to elucidate proper information on how chondrocytes dedifferentiation may be affected by the various aspect ratios on the surface^3^. Thus, interfacing anti-fouling surfaces with chondrocytes remains an important aspect in resisting dedifferentiation of chondrocytes by maintaining spheroid morphology.

**Figure 7:**
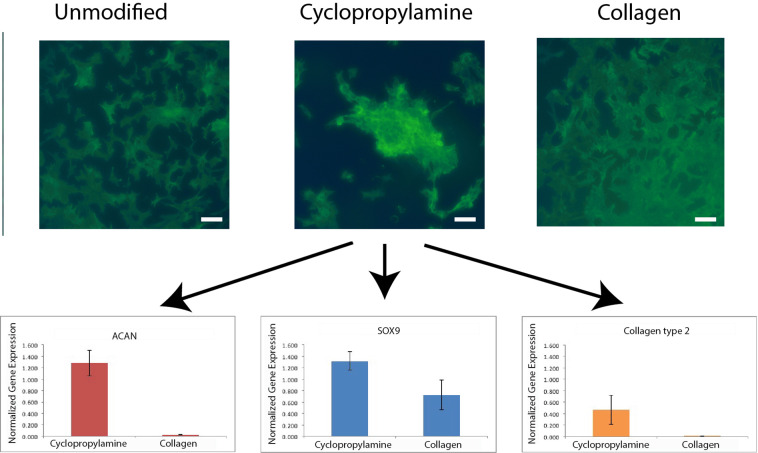
Confocal microscopy of phalloidin-stained P1 ACs cultured on Si substrates for 6 days. The quantification from the qRT-PCR of chondrogenic gene expression of P1 ACs cultured on Si substrates for 6 days were as shown below. White bar represents 20 μm.

We hypothesize that expansion of articular chondrocytes on cyclopropyl-functionalized surfaces, which had been shown to reduce intracellular assembly of focal adhesions, will allow greater retention of the chondrogenic spheroid phenotype during the expansion process. Primary articular chondrocytes (ACs) were isolated from minipigs and passaged once (P1) to obtain sufficient cells for experimental purposes. P1 ACs were cultured on unmodified, cyclopropylamine-functionalized, and collagen-functionalized Si substrates for 6 days until near confluence. Confocal microscopy of phalloidin-stained chondrocytes showed fibroblast-like cells with extensive filopodia/lamellipodia on both the unmodified and collagen-functionalized substrates, while cells cultured on cyclopropylamine were less spread and formed aggregates (Fig. 7). Gene expression analysis of chondrocytes expanded on cyclopropylamine had shown significant increase in chondrogenic markers *COL2A1*, *ACAN*, and *SOX9* compared to cells cultured on unmodified and collagen-functionalized surfaces, indicating that ring-strained surfaces indeed allowed better retention of the chondrogenic phenotype during chondrocyte expansion. Thus, ring-strained surfaces offers a unique non-chemical strategy for reducing focal adhesions and controlling cell differentiation, with particularly promising application in expanding chondrocytes so that they possess higher chondrogenic potential for cell therapy.

As a proof-of-concept, we also performed a 7-day incubation of cyclopropylamine grafting with commercially available Human Mesenchymal Stem Cells as shown in Fig. S8. After 7 days of prolong cell culture, we were able to observe similar anti-biofouling trends and this is highly encouraging as we had adequately demonstrated that our chemistries were highly stable and can resist cellular adhesion even for prolong periods of time.

## Discussion and Conclusion

It is unusual in existing literature that a ring-strained monolayer grafted on silicon surface can impede and discourage cellular adhesion. In this manuscript, we described this new surface modification and presented that as a proof-of –concept and we had subsequently shown that the antifouling effects from such surface modifications can inherently remove cell adhesion over a wide spectrum of different types, ranging from conventional immortalized cancer cells to even primary cell types such as mesenchymal stem cells and chondrocytes. From an academic standpoint, such an effect is unique and this help to open up newer avenues for surface modifications with the intentions of developing anti-biofouling thin films.

Traditionally, many groups had relied on thick hydrophilic surface grafts to attain anti-biofouling properties on many types of substratum. Yet, there had also been reports of hydrophobic surfaces possessing similar attribute as well. For instance, Bozukova *et al*. had reviewed on the issue of hydrophobic substrates that conferred cell repellency and suggested that this may be due to the adaption of unique structural configuration for surface absorbed proteins that is unrecognizable by incoming cells and hence the lower cellular adhesion^45^. The structural rearrangement of absorbed surface proteins had been well documented by Otsuni *et al*.^80^ using mixed SAM. Furthermore, when comparing mixed SAM on gold surfaces, Arima *et al*. had also observed that CH_3_/OH mixed SAM had the tendency to strongly attract serum albumin and this had prevented the subsequent absorption of cell adhesive proteins such as fibronectin etc to the surface^64^. From our cellular viability, microscopy as well as protein absorption studies (see Supplementary Fig. S4), we believed that our results had corroborated well with previous literature on hydrophobic surfaces, especially when our XPS had also revealed a mixture of both CH_3_ and surface OH (a result of steric hindrance on Si (111) surfaces). As many previous reports on using mixed SAM had often relied on large polymeric system to obtain similar wettability profile (~60–80°) as well as cell/protein repellency effect^81–83^, what that had set our work apart is the fact that grafting of short cyclic monolayer is already sufficient to obtain similar findings. Interestingly, while 1,7 octadiyne surfaces in our work had also shown similar hydrophobicity profiling, we did not observe high anti-biofouling effects. This may be also due to the fact that a mixed surface of CH_3_/OH as reported by Arima *et al*. may require periodical segregation of both moieties while aliphatic 1,7 octadiyne may have been grafted in patch-like domains on the surface and thus loses the mixed SAM properties. On the other hand, due to the steric effects of the cyclic rings, we were able to form better mixing of both OH and CH_3_ across the surface but this would require further studies in the future.

We have demonstrated in this paper that not only was our cyclic monolayers on silicon surfaces anti-adhesive in nature, the stability and the anti-adhesive potential of these monolayers was substantiated by the prolong chondrocytes culture for up to a period of 6 days. In fact, the incorporation of MSC incubated over a period of 7 days had also revealed similar results and our current undertaking in our group had in fact revealed a reduction of overall cell number for MSC for up to 28 days (unpublished data). So far in this work, we had included a wide variety of cell types, ranging from immortalized cell lines to primary cell culture and our results had shown similar trends in terms of producing antifouling effects regardless of cell type. From our data reported here, we had noticed that these anti-adhesive effects were highly comparable to those from conventional surface pegylation of a 6–9 ethylene glycol unit on silicon surface and the quantification of a series of different focal adhesion proteins had strongly suggested that these cyclic monolayers were able to discourage the extension of the cytoskeleton of the cells even upon surface adhesion. In fact, this effect was very much dependent on the cellular morphology, i.e. the more elongated and extensive the cells were, the more restrictive these monolayers could act upon.

While there is a wealth of information gathered from this report and we had also demonstrated the stability of the monolayer over prolong periods of incubation, there will certainly be room for investigation especially pertaining to the precise mechanisms by which such ring-strained monolayer are acting on the adhesion behaviour of these cells. For instance, we are curious how this chemistry may be exported to other substrate types such as on gold or softer polymeric materials. Current reaction model using thermal reaction may not exactly work with other substrates although the reason for doing so in this paper is on the basis of the material that had been chosen here, namely silicon surfaces. Certainly, the reactions on other materials such as gold may involve using milder chemical modification process although it is hypothesized that these materials having similar distal ends in terms of cyclic monolayers that may also induced similar traits. Work is currently undertaken in my group to help expand on these lines of thoughts and to better elucidate the precise mechanisms revolving around how these cyclic monolayers in fact interact with cells. For the purpose of clarity, this work as reported here represent a preliminary proof-of-concept on this unique chemical strategy. Nonetheless, in view of the results reported here, it is highly possible that these observations could help reshape some of the fundamental concepts regarding cell-surface interactions.

## Supplementary information

Supplementary information.
