# Supplementary material for: Anti-Adhesion Behavior from Ring-Strain Amine Cyclic Monolayers Grafted on Silicon (111) Surfaces
Source: Sci Rep. 2020 May 29;10:8758. doi: 10.1038/s41598-020-65710-w (PMC7260185; doi:10.1038/s41598-020-65710-w)
Supplement: Supplementary file 1 — Supplementary information. [file 41598_2020_65710_MOESM1_ESM.docx]

Supporting Information

Anti-Adhesion Behavior from Ring-Strain Amine Cyclic Monolayers Grafted on Silicon (111) Surfaces

***Jing Yuan Ching^a^, Brian. J. Huang^b,c^, Yu-Ting Hsu^a^ and Yit Lung Khung^a*^***

**
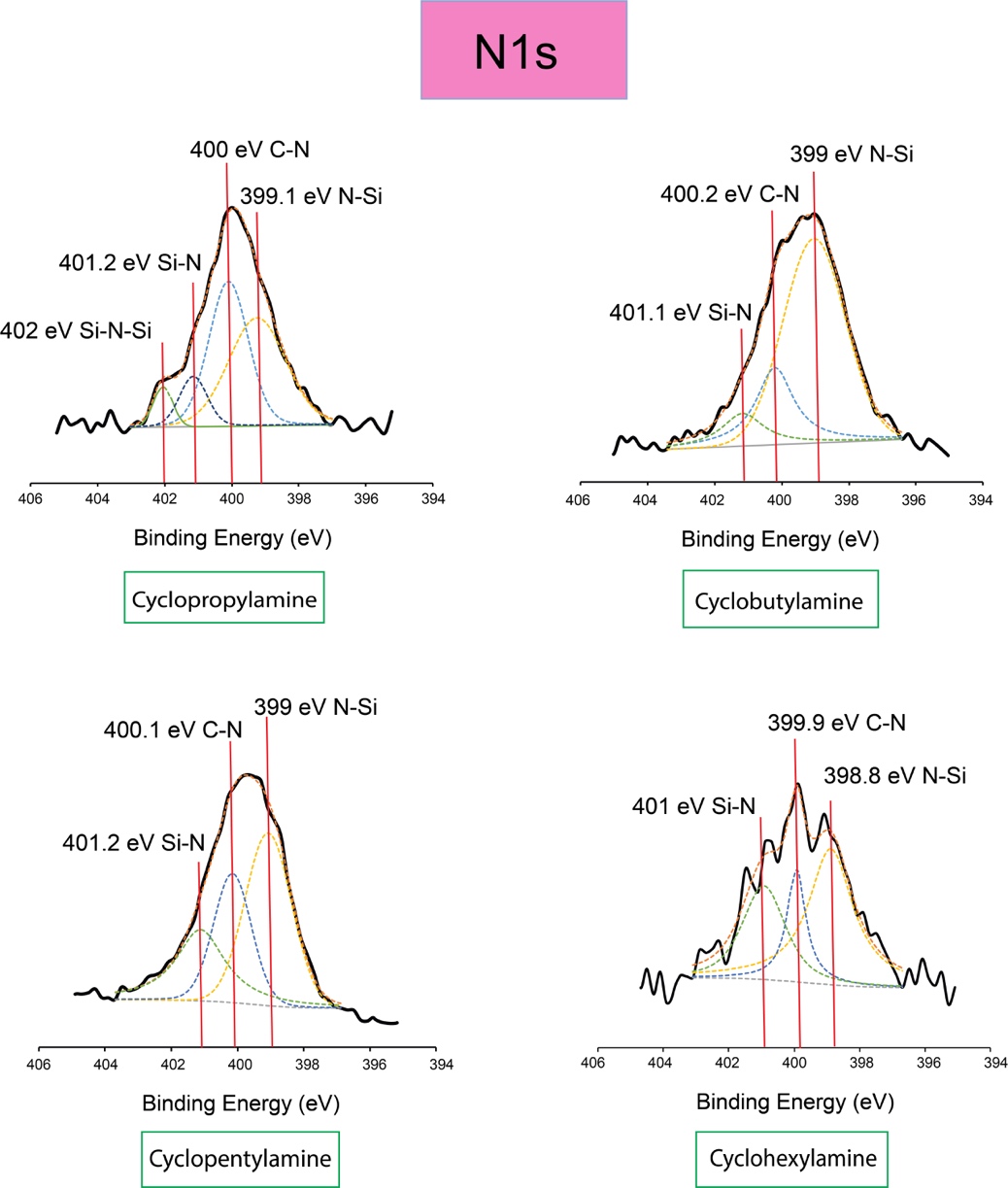
**

Figure S1. XPS N1s for Si (111) surfaces modified with the various cyclic molecules

**
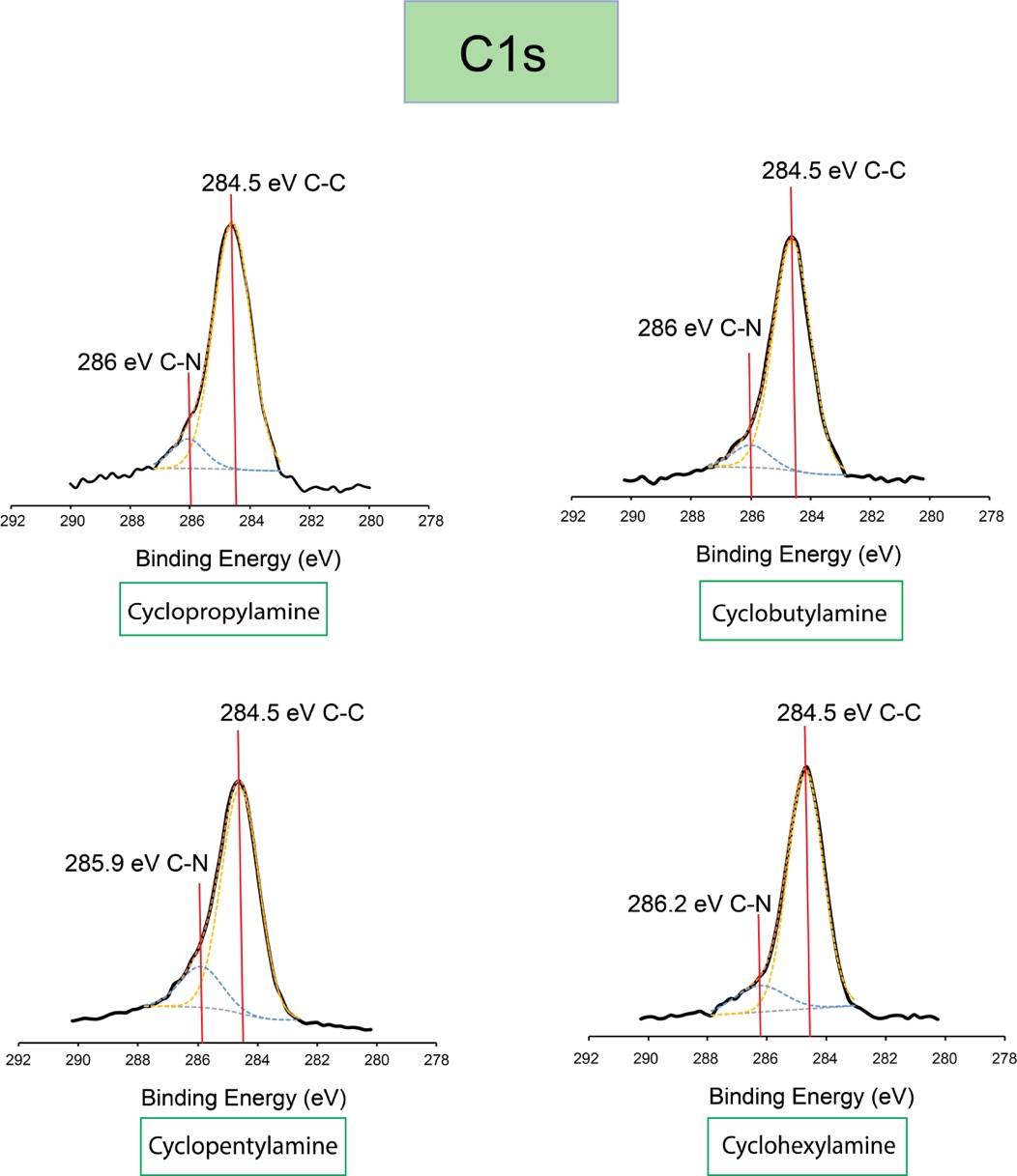
**

Figure S2. XPS C1s for Si (111) surfaces modified with the various cyclic molecules

**
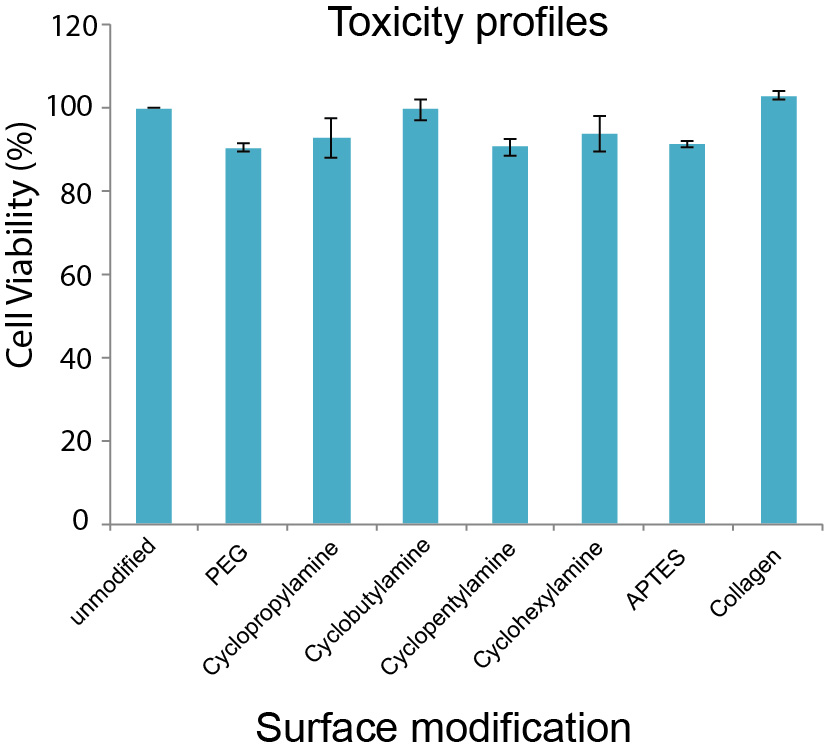
**

Figure S3. Toxicity profile of the surface modifications. MDA-MB 231 cells were incubated alongside in culture well containing the various surface modification on the silicon (111) substrate and the overall viability profile was collected after 24 hours of incubation

**
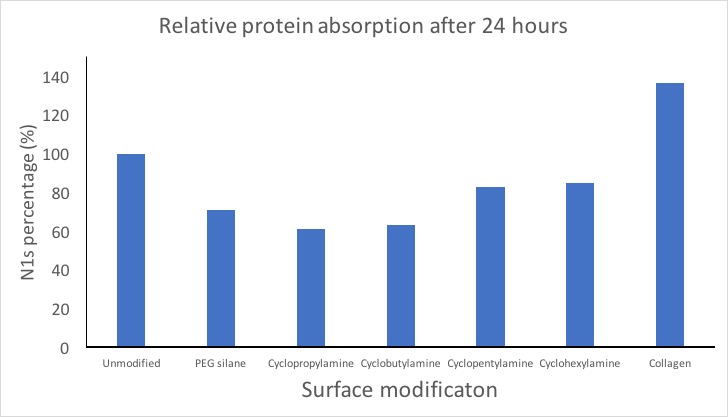
**

Figure S4. Relative FBS absorption on the various surfaces after 24 hours of incubation. The values were determined from N1s analysis.

**
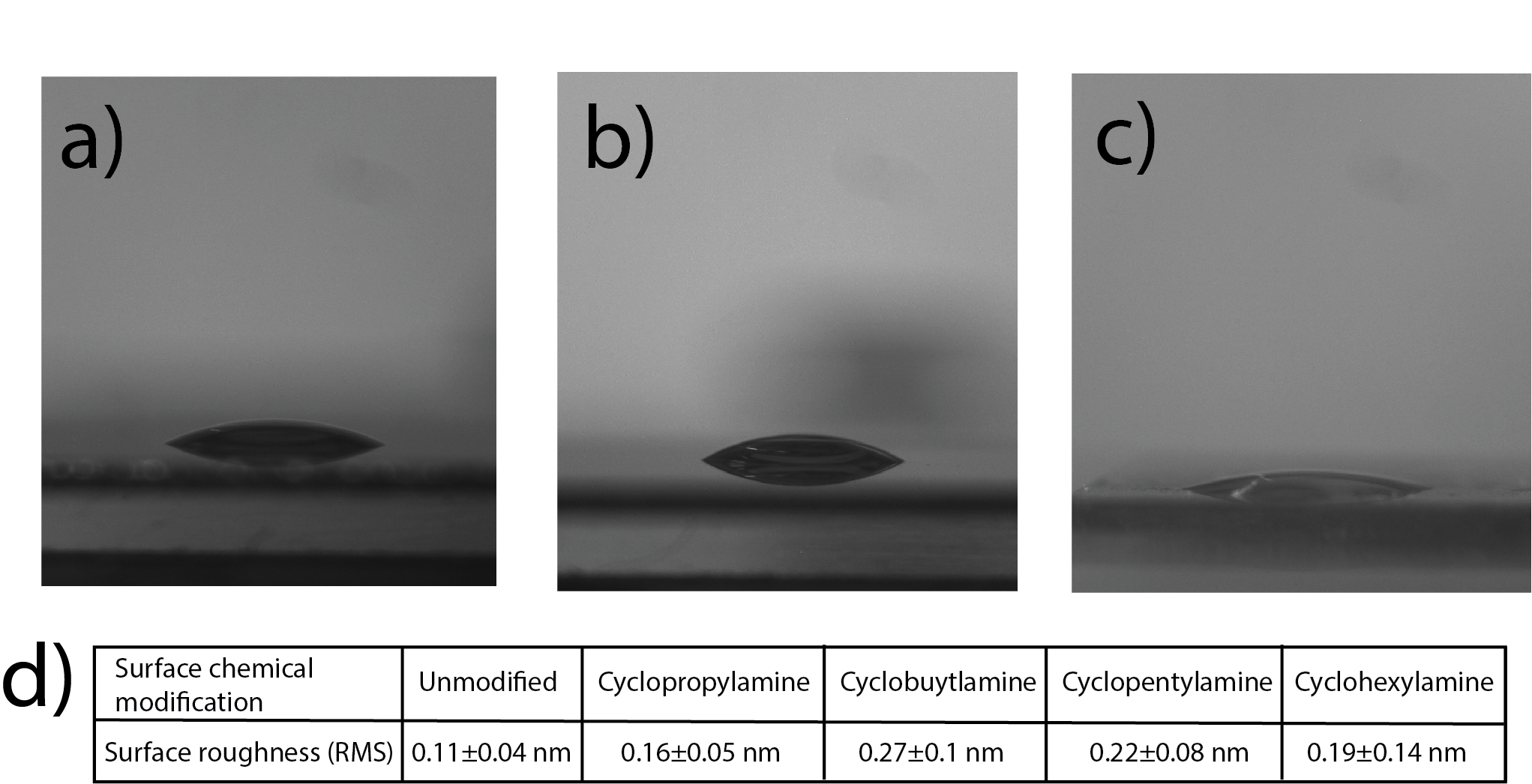
**

Figure S5. Contact angle images of (a) APTES modified surfaces, (b) PEG functionalized surface and (c) collagen coating. The surface roughness of the cyclic monolayers modified silicon surfaces are as tabulated below (d).


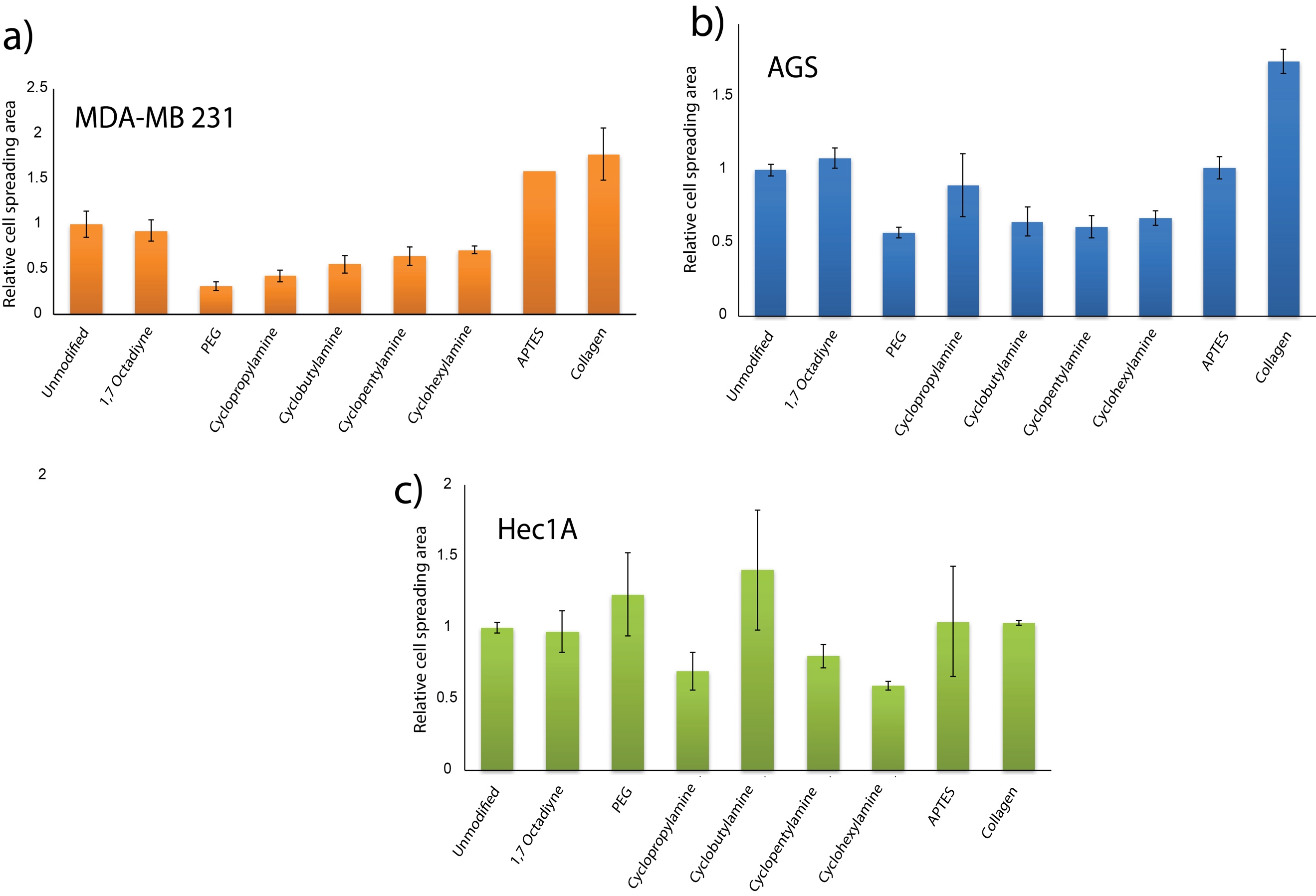


Figure S6. Cell spreading area as calculated for (a) MDA-MB 231, (b) AGS and (c) Hec1A after 24 hours of incubation on the various surfaces


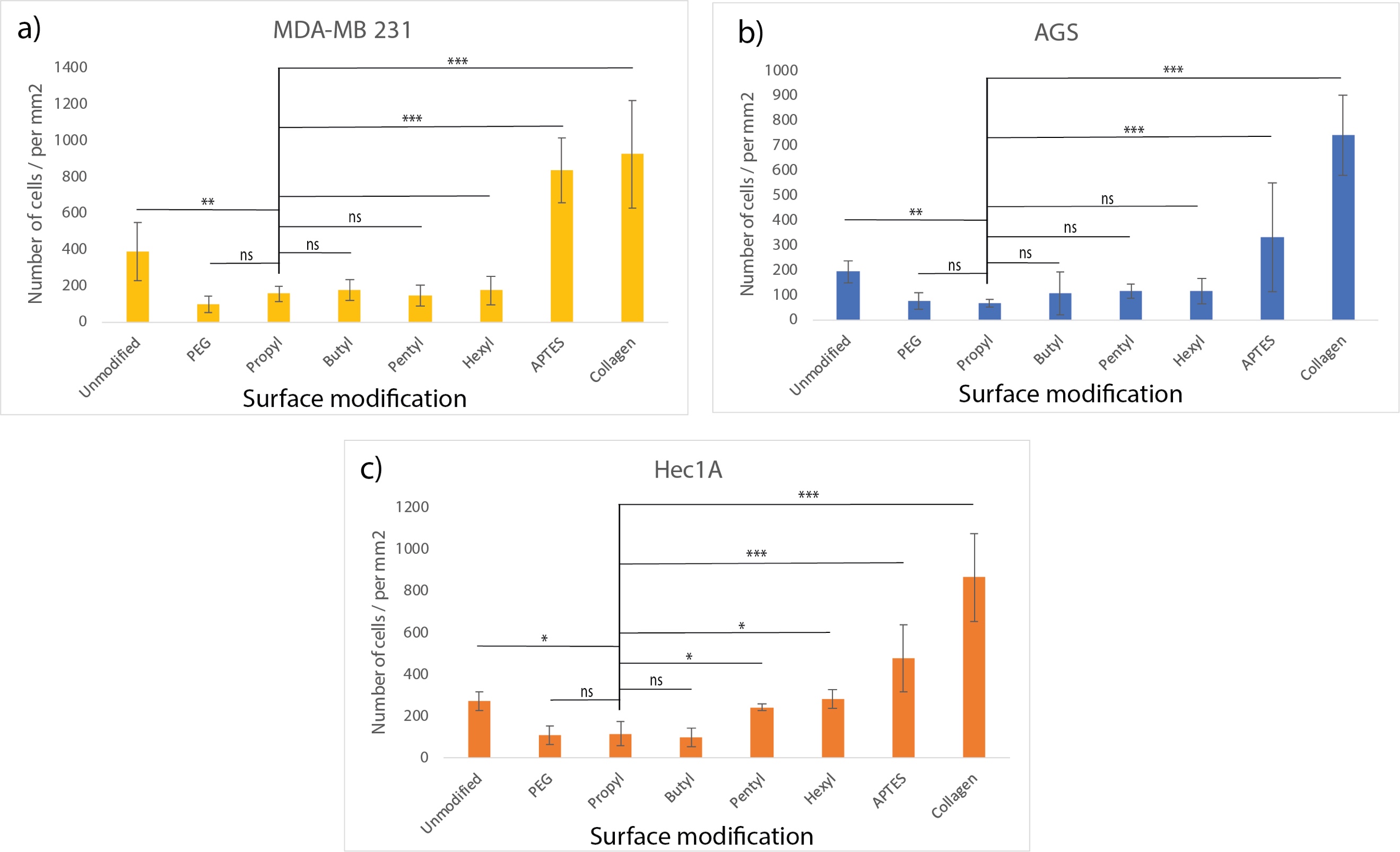


Figure S7. Cell counts on the various surface modifications for (a) MDA-MB 231, (b) AGS and (c) Hec1A after 24h of cell culture.


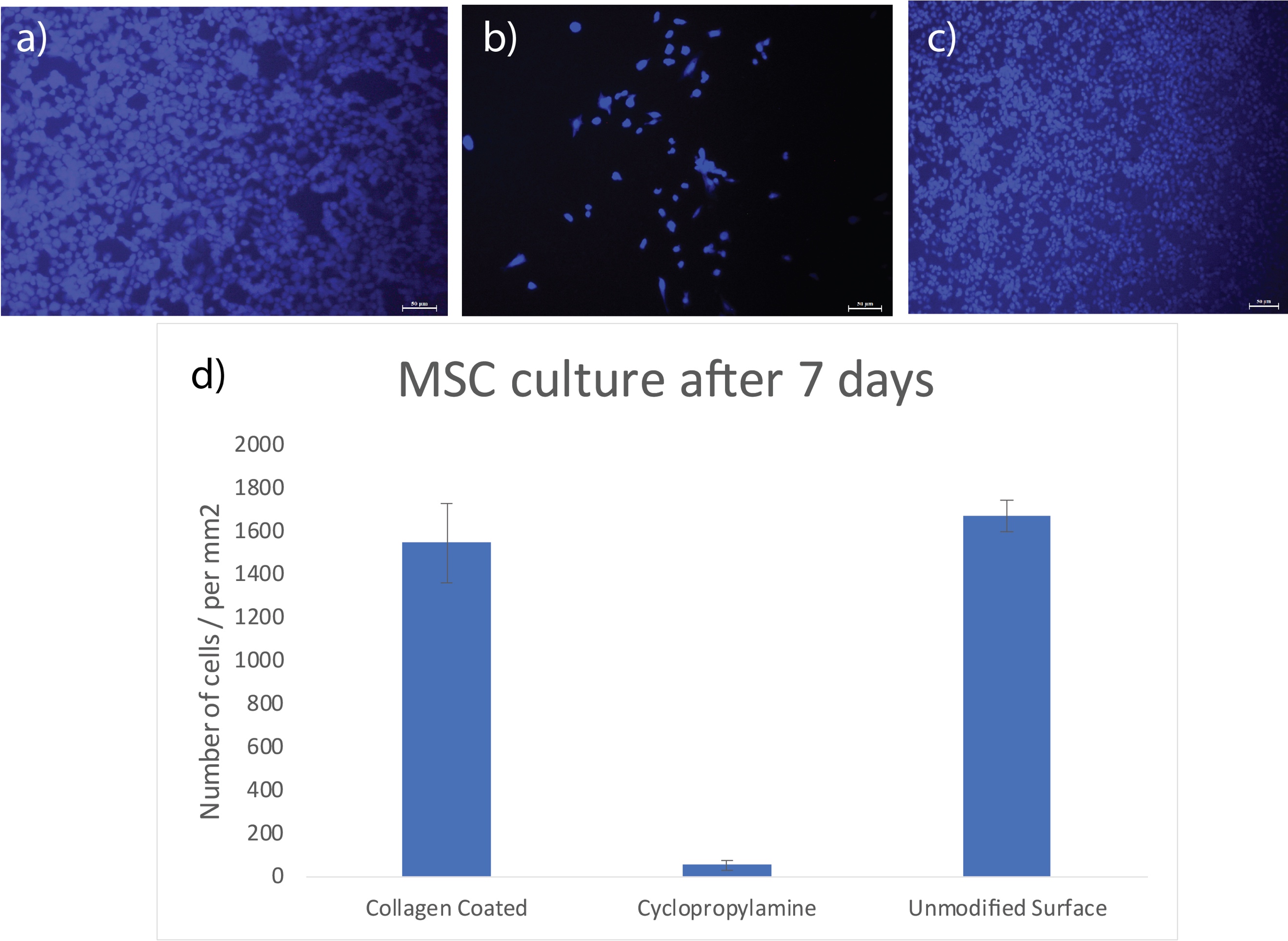


Figure S8. Human Mesenchymal Stem cells incubated over a period of 7 days on (a) unmodified silicon surfaces, (b) cyclopropylamine surfaces and (c) collagen coating. The cell count of these surfaces were also measured as shown in (d)
